# Treatment-seeking behaviour for malaria in children under five years of age: implication for home management in rural areas with high seasonal transmission in Sudan

**DOI:** 10.1186/1475-2875-5-60

**Published:** 2006-07-22

**Authors:** Elfatih Mohamed Malik, Kamal Hanafi, Salah Hussein Ali, Eldirdieri Salim Ahmed, Khalid Awad Mohamed

**Affiliations:** 1 National Malaria Control Programme (NMCP), Federal Ministry of Health, P.O. Box: 1204, Tel: +249 183 769579, Fax: +249 183 770397, Khartoum, Sudan; 2 Health Education Executive Director, National Health Promotion Directorate, Khartoum MOH, Sudan; 3 Behavioural surveillance unit, HIV/AIDS Programme, Khartoum MOH, Sudan; 4 College of Medicine, University of Juba, Sudan; 5 Alder Hey Children's Hospital and University of Liverpool, Liverpool, UK

## Abstract

**Background:**

Effective management of malaria in children under the age of 5 requires mothers to seek, obtain, and use medication appropriately. This is linked to timely decision, accessibility, correct use of the drugs and follow-up. The aim of the study is to identify the basis on which fever was recognized and classified and exploring factors involved in selection of different treatment options.

**Methods:**

Data was obtained by interviewing 96 mothers who had brought their febrile children to selected health facilities, conduction of 10 focus group discussions with mothers at village level as well as by observation.

**Results:**

A high score of mothers' knowledge and recognition of fever/malaria was recorded. Mothers usually start care at home and, within an average of three days, they shift to health workers if there was no response. The main health-seeking behaviour is to consult the nearest health facility or health personnel together with using traditional medicine or herbs. There are also health workers who visit patients at home. The majority of mothers with febrile children reported taking drugs before visiting a health facility. The choice between the available options determined by the availability of health facilities, user fees, satisfaction with services, difficulty to reach the facilities and believe in traditional medicine.

**Conclusion:**

Mothers usually go through different treatment option before consulting health facilities ending with obvious delay in seeking care. As early effective treatment is the main theme of the control programme, implementation of malaria home management strategy is urgently needed to improve the ongoing practice.

## Background

Malaria in Sudan is a leading cause of morbidity and mortality with an annual estimate of 7.5 million cases and 35,000 deaths. This represents 50% and 70% of the WHO/Eastern Mediterranean Region cases and deaths respectively [1]. Malaria is the main cause of fever in children less than 5 years of age. Early diagnosis and appropriate treatment are essential to reduce morbidity and mortality related to malaria among this group [2,3]. This is always influenced by several factors especially in rural areas. Treatment-seeking behaviour is related to cultural beliefs about the cause and cure of illness [4]. The choice of treatment source was found to be influenced by accessibility, disease type and severity, patient's gender and parents' educational level [5-7]. Attitude towards providers was also an important factor [8]. Patients are more likely to start with self-treatment at home as this allows them to minimize expenditure [9-11]. In Sudan, two third of the patients in rural areas administer anti-malarial drugs without laboratory confirmation with preference of injectable forms [12].

However, with the shift to artemisinin-based combination therapy (ACTs) in Sudan, the issue of treatment-seeking was considered as the cost of drugs is clearly higher compared to the cost of chloroquine. This study aimed to identify the basis on which fever was recognized and classify and explore factors involved in selection of different treatment options.

## Subjects and methods

### Study setting

This cross-sectional community-based study was conducted in the west of the Sudan (Kordofan/Umadara Area). The population in the area is around 75,000, distributed over 43 villages, with some nomads. The area was selected out of more than 100 rural districts in Sudan as it was perceived by the research team to meet many criteria. It has been one of many unrest areas in Sudan for years; different ethnic groups live in the area and so are expected to reflect different opinions and it is one of the areas with a nomadic population. Malaria in the area is meso-to hyperendemic with high and very long seasonal transmission (May – November).

### Sample size

A total of 10 villages were found to be convenient to answer the study questions related to malaria treatment-seeking behaviour at the community level. Focus group discussions (FGDs) with 10 groups of mothers (average of 11 women per FGD) were carried out in the selected villages. A sample of 96 mothers of those who attended to the health facilities with a febrile child during the study period were interviewed (calculated using EpiInfo 2002 based on 50% prevalence and 95% CI).

### Sampling technique

Villages were selected randomly from a sampling frame using the random number list of EpiInfo package. Mothers in each village were arranged for FGDs. Selection of mothers for interview at each health facility was carried out by the team leader. The team leader reviewed the records to estimate the expected mothers during the study period at each facility, then divided the total sample (96 mothers) into groups, distributing the groups to the different facilities, based on the expected attendance (probability proportional to size). The data collectors stayed at the facility interviewing every mother with febrile child until the target number was reached.

### Data collection

Three trained social workers, using the pre-coded and pre-tested questionnaire, interviewed the required sample size from mothers who had brought their febrile children to 15 selected health facilities. Also they conducted FGDs with mothers at village level using the FGDs guide. The DILO (a day in the life of villagers) methodology was adopted also [13]. This implies the presence of the team designated to collect the data in each village from morning to evening. The required work was divided between the team members. A minimum work was required from each but while they were moving in the village they were free to make use of any event (e.g a febrile child, death,...).

### Data management and analysis

By the end of each day, the team leader arranged for a meeting to compile the data and to keep it for summarizing later. Each day, the team made use of the lessons learned in the previous working day. Qualitative data were analysed using MAXQDA software. Quantitative data was processed and analysed using SPSS computer software. Appropriate test were used to test for significance (x2, t-test)

### Ethical considerations

This study was approved by the Ethical Review Committee (Federal Ministry of Health). In each village the study started after receiving permission from community leaders.

## Results

### Recognition, classification and response to fever

The 96 mothers interviewed have a mean age (SD) of 26.1 (5.9) years, 55 (58.3%) were illiterate, 91 (94.8%) were solely housewives or housewives and farmers and 90 (93.8%) have 1–3 children less than 5 years. Based on the type of housing, family source of income and presence of domestic animals, 79 (82.3%) of mothers have low to moderate socioeconomic status.

Fever was recognized by 68 (70.8%) and 16 (24.0%) of mothers as '*hot body*' or as a syndrome including '*hot body*' and other symptoms *'headache, restlessness...' *respectively. Malaria was correctly defined as 'fever or fever with other symptoms or signs' and was recognized as a common cause of fever followed by chest infection. Fever was considered as a dangerous feature leading to complications or to death as revealed by focused group discussions (FGD). Fifty-eight of interviewed caregivers (60.4%) responded to fever by seeking advice from sources other than health facilities initially; of those (90%) offered their child a drug. The mean duration before attending a health facility was 67.8 hours. The main reason cited by mothers for their attendance to health facilities was *'deterioration of the child condition'*. The decision for seeking treatment at health facility was the parent's decision (Table 1).

**Table 1:** Mothers' response to fever (n = 96 unless indicated)

**Variables**	**Frequency (%)**
Mothers recognized fever as "hot body" or "hot body with others symptoms or signs"	68 (70.8%)
Mothers defined malaria as "fever or fever with other symptoms or signs"	84 (87.5%)
Mothers blamed malaria for the child current fever	38 (40.0%)
Mothers believed that the common cause of fever in the area is malaria	76 (79.2%)
Mothers seeking advice from any source before coming to health facilities	58 (60.4%)
Mothers give the child any sort of care before coming to health facilities	49 (51.0%)
The care given to the child was drugs (n = 49)	44 (89.8%)
Village is the source of the care given (n = 49)	43 (87.8%)
Duration (in hours) of response from the initiation of fever (Mean ± SD)	67.8 ± 41.3
Mothers now in this facility because child condition deteriorated	93 (96.9%)
Decision for coming here was the decision of:	
• Mother	41 (42.7%)
• Father	17 (17.7%)
• Mother/father	33 (34.4%)
• Others	05 (05.2%)

### Most common options for treatment

The preferred and actual practice related to treatment of fever in children is detailed in Table 2. Four main treatment options available for any patient in the area: consulting health workers, traditional medicine, use of herbs and self-treatment. FGD showed that, although it was not mandatory, people when getting ill think about consulting health workers at the health facilities or at health workers home or if the patient couldn't move they ask the health worker to visit him at home. During rainy season, one of the patient's relatives visits the health worker at home and describes the patient symptoms and accordingly the drug is given to this relative for the patient.

**Table 2:** preferred and actual practice related to treatment of febrile children

**Variables**	**Frequency (%)**
Mothers **prefer **to seek advise from:	
• Health workers	78 (81.3%)
• Grandmothers, grandfathers, neighbours	07 (07.3%)
• Village volunteers	04 (04.2%)
• Others	03 (03.1%)

Mothers **actually **seek advise for this event from:	
• Health workers	81 (84.4%)
• Grandmothers, grandfathers, neighbours	07 (07.3%)
• Village volunteers	00 (00.0%)
• Others	04 (04.3%)

Self-treatment was common. Reasons stated include: their ability to recognize malaria, the cost of travel and, on some occasion, the lack of health care facilities. Sometimes parents seek advice from other community members, if they agreed that the sickness is malaria they administer antimalarial drugs. People obtain drugs, commonly chloroquine, aspirin and paracetamol from private pharmacies or drug stores in nearby villages or cities. Dosage is decided based on people's experience. People usually start with self-treatment and then they look for treatment in nearby facilities. FGD with mothers and visits to 4 sick children- found to be sick at village level- confirmed what mentioned above.

### Factors involved in the selection between different treatment options

Two major points mentioned during FGDs affecting health-seeking behaviour. When the child condition deteriorated, with *'high fever, inability to stand or walk, refusal to feed, loss of consciousness, yellowish sclera, severe diarrhoea and repeated vomiting'*, there is an urgent need for health worker consultation. On the other hand, any condition what so ever its severity if started at any time at night the child has to wait till the morning. Other reasons include: low coverage and/or performance of health facilities, the expected cost and frequent use of traditional medicine and herbs. Seeking help from health personnel and not from other options has no relation to the parent's or child's age or gender. However, there was significant association between father education and consulting health workers within 24 hours of the onset of fever (*P < 0.05*).

## Discussion

Effective management of malaria requires the consumers and the care- givers, seek, obtain, and use drugs appropriately [14]. This is linked to timely decision, accessibility, correct use of the drugs and follow-up after prescription.

Malaria in children under 5 years requires caregiver's early recognition and classification of fever. In the present study, fever and malaria were defined correctly by the majority of caregivers and malaria was identified as a main cause of fever. These findings have been shown to be the key to intervention in rural Ghana [15]. The study results reflected mother's good knowledge about malaria, its transmission and prevention; as in other parts of Africa [16]. Furthermore, they identified that *high fever, inability to stand or walk, refusal to feed, loss of consciousness, yellowish sclera, severe diarrhoea and repeated vomiting *were the features if malaria episode evolved into a more serious situation (severe malaria) and that requires urgent treatment at health facilities. These findings were consistent with similar studies carried out in Sri Lanka [17] and in other parts of Africa [9,18].

Four treatment options were available for a febrile child in the area. Two included the use of drugs (consulting health workers and self treatment). The other two (traditional medicines and herbs) were deeply rooted. Accessing care from a variety of sources is a common practice in malaria endemic areas. A study in Philippine [19] showed availability of six treatment choices for families ranging from 'not doing any thing for the patient' to 'treatment with drugs based on formal prescription'. Sources of health care identified in Uganda, included public health institutions, private practitioners, traditional healers and self-treatment [20].

Treatment-seeking behaviour was comparable between villages in the study area. Commonly people start care for a febrile child at home with what available (herbs, remaining drugs, drugs from shops, tepid sponging), when there is no response or if the condition deteriorates then they seek advice from health personnel. Medication at home before moving to health facilities was reported also in Tanzania [21]. A study in Kenya [9] showed that moving from different options determined by duration of sickness, its intensity and the expected cost. As stated by others, the delay in seeking care at health facilities level was related to existence, accessibility, satisfaction [20] and cost [15] of service, as well as satisfaction with traditional medicine and herbs. However, two factors affecting early consultation were actually leading to contradicting results: firstly, severely ill child need urgent consultation and hence short duration and secondly, appearance of illness at night deter the child from health facilities care waiting for the sun to rise and hence prolong the duration. These and other barriers were recognized by other researchers. For instance, Nuwaha F. in Uganda [20] added long waiting time, health workers abusing patients and being given tablets instead of injections as important barriers. Hill Z. et al in Ghana [15] considered financial access as a major barrier to care seeking.

Self-treatment and traditional medicine are habitual among the population of the study area. Similar finding had been reported in other parts of Sudan [12] and in Tanzania [16]. In the present study, seeking health care at health facilities is predominantly decided by mothers, this in contrast to what mentioned by Nsungwa-Sabiiti et al in Uganda [18] where mothers decide only when treatment is uncharged.

In Sudan, more emphasis for delivery of antimalarials including ACTs is given to health facilities. However, as shown by this study, in situations where coverage with health facilities is low, promoting adequate case management practices at the community level appears necessary. It has been documented [22,23], through training of mothers and availing adequately packaged drugs, mothers could recognize malaria and as well give appropriate treatment at home and, by doing so, reduce the incidence of severe disease and thus mortality.

## Conclusion and recommendations

This study explores the requirements of a successful home management strategy [24,25]. The implementation of such strategy should take into account the ongoing treatment options in the area including private drug dispensers. A volunteer nominated from each village (called malaria control assistant -MCA) can be trained, equipped and linked with health facilities in each area. Pharmacies at health centres or hospitals can be used to store drugs for the whole area over the malaria season to ensure the quality and to distribute regularly to MCAs. Severe malaria cases identified by MCAs can then be referred to the health facilities after receiving one dose of artesunate suppository.

## Authors' contributions

EMM carried out the field work, analysed the data and drafted the manuscript.

KH participated in the design of the manuscript and revised the manuscript and given the final approval to be published. All authors read and approved the final manuscript.
